# The role of emotion in the dyad inversion effect

**DOI:** 10.1371/journal.pone.0219185

**Published:** 2019-07-02

**Authors:** James W. A. Strachan, Natalie Sebanz, Günther Knoblich

**Affiliations:** Central European University, Budapest, Hungary; Universita degli Studi di Udine, ITALY

## Abstract

When observing two individuals, people are faster and better able to identify them as other people if they are facing each other than if they are facing away from each other. This advantage disappears when the images are inverted, suggesting that the visual system is particularly sensitive to dyads in this upright configuration, and perceptually groups socially engaged dyads into a single holistic unit. This dyadic inversion effect was obtained with images of full bodies. Body information was sufficient to elicit this effect even when information about head orientation was absent. However, it has not been tested whether the dyadic inversion effect occurs with face images and whether the emotions displayed by the faces modulate the effect. In three experiments we obtained robust dyadic inversion with face images. Holistic processing of upright face pairs occurred for neutral, happy, and sad faces but not for angry and fearful face pairs. Thus, perceptual grouping of individuals into pairs appears to depend on the emotional expressions of individual faces and the interpersonal relations they imply.

## Introduction

Humans can decode a great deal of social information at a glance. When observing individual others, people make fast, consistent [1,2], and accurate [3] (although see [4]) inferences about their personality and internal emotional or mental states [5]. The capacity to process social information has been explored at the level of perceiving individual people, but there has been limited research on how people view the relationships and interactions of others from an allocentric perspective–that is, how they process information about social systems that they are not directly a part of. Although mechanisms for decoding social information relating to hierarchies and alliances in allocentric contexts have been addressed [6], this research has focussed on quite abstract inferences based on prior knowledge of the individuals involved. There has been comparatively little work looking at whether there may be similar early visual mechanisms for processing groups of people as for processing individuals in isolation.

Recent evidence suggests that people can process relational information about two individuals in a similarly privileged way to how information about single individuals is processed. Papeo, Stein and Soto-Faraco [7] presented the first evidence of this using a classic experimental technique: body inversion. Perceptual processing of bodies is typically fast and accurate, but it is severely disrupted when images of bodies are presented vertically inverted–a disruption that is not seen for non-body objects [8,9]. This disruption is thought to reflect the visual system’s sensitivity to bodies in a canonical upright configuration, where the spatial arrangement of component features is necessary for it to be processed as a body. Papeo et al. adapted this effect by presenting participants with images of two bodies that were either oriented towards each other or away from each other. They found that for dyads the classic inversion effect was only seen for pairs of bodies that were facing each other and was absent for pairs that were facing away from each other. This effect was only observed with bodies, and not with non-body objects (chairs) that also contained a ‘front’ and ‘back’ and were arranged in a facing or non-facing configuration.

Papeo et al. [7] interpret this as evidence of perceptual grouping of bodies that are facing each other. They suggest that bodies oriented such as to suggest that they are socially engaged are treated as a single perceptual unit, while disengaged pairs are not grouped and so must be processed in a more piecemeal way. This reflects an early perceptual mechanism where the visual system is particularly sensitive to this configuration and privileges processing of engaged (facing) pairs of bodies. This perceptual grouping mirrors effects seen with functionally related objects, where people are also faster to process objects that are spatially configured to reflect their relationship (e.g. a teapot and a teacup are grouped together and processed more efficiently if the spout of the teapot is oriented towards the teacup, [10–13]). Perceptual grouping of engaged dyads may thus indicate that participants expect this configuration to have some functional relevance (in this case, reflecting a social interaction or relationship).

Vestner, Tipper, Hartley, Over, and Rueschemeyer [14] have also investigated this two-body inversion effect and found that engaged pairs of bodies are detected faster in a visual search paradigm when they are presented upright than when they are inverted, while there is no such disruption for disengaged pairs. They ruled out an explanation based on visual symmetry, showing that the effect is present even for adult-child pairs, which are visually asymmetrical, but absent for highly symmetrical non-body objects such as dressers. Papeo, Goupil and Soto-Faraco [15] also used a visual search paradigm to show that engaged dyads are detected more easily as targets and dismissed more efficiently as distractors than disengaged dyads. Furthermore, local processing of individual bodies was compromised when those bodies appeared in facing dyads. This was interpreted as a consequence of grouping those bodies into a dyadic unit: it may be more difficult to find a particular individual appearing in an engaged dyad than a disengaged dyad, because the visual system treats the dyad as the unit of interest rather than individuals.

To date, evidence for holistic perceptual processing of dyads has been restricted to full body images. Papeo et al. [7] found that this perceptual grouping effect (a greater inversion effect for engaged dyads than disengaged) persisted when the heads of the dyads were blurred. More recently Papeo and Abassi [16] showed that blurring the head of bodies does not affect the signature of the dyadic inversion effect. Thus, there is evidence that head orientation is not necessary in eliciting the dyad inversion effect. However, it has yet to be seen whether information from the head or face is sufficient to trigger perceptual grouping of dyads. Like bodies, faces also show a clear inversion effect [17,18], which relies on disruptions to configural processing in a similar way to both the single-body and body dyad inversion effects [19]. This raises the question whether social relations conveyed through face images alone lead to a similar dyad inversion effect.

We use the inversion effect as an indirect measure of this proposed perceptual grouping mechanism, given that perceptually grouped and ungrouped stimuli should be affected differently by inversion. The classical inversion effect is typically taken as a measure of visual sensitivity to a particular configuration of stimulus features–the specific configuration of facial and body features is as important or more important in recognition than the individual features themselves [7,8,18,19]. Perceptual grouping of dyads is driven by the spatial configuration of two individual bodies (dyads are grouped if the bodies are socially engaged, but not if they are disengaged), and a key assumption is that this spatial relationship is as important or more important in recognising a dyad than the component bodies. As such, inversion effects can be used to indirectly measure perceptual grouping by looking at the *pattern* of inversion effects. The prediction of a perceptual grouping mechanism is that the visual system is more sensitive to the engaged configuration of dyads than to the disengaged configuration. In particular, the engaged configuration should be *especially* disrupted by inversion. That is, in this study we do not look for isolated inversion effects but for a particular RT signature characterised by a stronger inversion effect for engaged dyads and an attenuated (or absent) inversion effect for disengaged dyads.

We report three experiments that aim to replicate and extend the dyadic inversion effect with face images alone. Using faces, we explore whether the emotional expressions of faces, and the apparent relation conveyed by these emotions, can influence the extent to which dyads are grouped into single perceptual units. Facial expressions are decoded quickly [20], automatically [21] and independent of top-down attention [22], and they can not only inform an observer of an individual’s internal mental state but also affect how one processes the relationship between an individual and their environment–objects that are looked at by another person acquire affective evaluations that reflect the emotional expression of the gazing individual such that objects that are looked at with a smile are liked more, while objects that are looked at with disgust are disliked [23,24]. It remains to be seen whether the contextual relevance of emotional expression can also affect the early perceptual grouping mechanism responsible for the dyad inversion effect.

Processing facial expressions of emotion has been investigated with face inversion effects and the main finding was that explicit categorisation of facial expressions of emotion is compromised when faces are presented in an inverted orientation. However, the results of previous studies also show some inconsistencies. While some studies find that all emotions are subject to the same inversion effects [25], other studies have found different effects of inversion on categorisation of different emotions [26–28]–mainly that the easier or more discriminable an expression is when upright, the smaller the inversion effect [29].

Emotion discrimination, unlike identity discrimination, appears to be largely driven by particular facial regions such as the mouth and eyes–when these regions are left upright in a face that is otherwise inverted, a manipulation known as the Thatcher Illusion, recognition of happy, neutral, and fearful expressions is better than when these regions are locally inverted [30]. This means that those emotions associated with easily discriminable changes in these regions (such as smiles for happiness) are more resistant to inversion, while inversion has a large disruptive effect on perception of anger, disgust [29,31] and sadness [28].

If the dyadic inversion effect reflects the visual system’s sensitivity to a particular configuration because it is diagnostic of the social relationship between these individuals, then emotions suggesting a positive relationship (e.g., two faces looking at each other and smiling) may result in a larger inversion effect than emotions implying a negative relationship (e.g., two faces looking at each other with angry expressions) because positive allocentric interactions may imply a group or affiliation that may be adaptive to join. On the other hand, a sensitivity to threatening or negative third-party interactions may also serve some adaptive function, as it may be advantageous to know if two people are likely to grow violent [32,33], in which case we may expect more evidence for a dyadic inversion effect for expressions that imply a negative relationship than a positive one.

## Experiment 1

In our first experiment we aimed to replicate the dyadic inversion effect with faces. Our prediction was that two faces looking towards each other should be subject to an inversion effect such that responses are faster and more accurate when the images are presented upright rather than inverted. This inversion effect should be attenuated or even absent for dyads looking away from each other, as being socially disengaged disrupts holistic processing of the dyad.

For this experiment we used faces posing three different facial expressions of emotion: neutral, happy, and angry. We predicted that we would replicate the dyadic inversion effect with neutral faces, as Vestner et al. [14] showed effects with bodies showing neutral body postures. If emotion plays no role in perceptual grouping of faces, then we should see similar dyadic inversion effects for all expressions. However, specific emotions might disrupt perceptual grouping, as the expressions of individuals change the apparent relationship of socially engaged pairs (faces looking towards each other). In this case we may expect a different pattern of inversion effects for happy or angry faces, as these emotions convey different types of information about the social relationship between the two individuals.

### Materials and methods

#### Participants

Using the estimated effect size from Vestner et al.’s [14] first experiment (which included a 2x2 design tested on RT data that was similar to the current study; f = 0.33), a power analysis with G*Power v3.1.9.2 estimated that 15 participants would be necessary to achieve 80% power. In order to achieve full counterbalancing we aimed to recruit 16 participants for every between-subjects condition.

We recruited 52 participants for this study, but due to high error rates on catch trials (details below) we had to exclude 4 participants, leaving a total of 48. There were 16 participants for each emotion (neutral: 8 female, M_age_ = 25.56yr; happy: 14 female, M_age_ = 23.31yr; angry: 11 female, M_age_ = 23.31yr). Participants were recruited through the Central European University SONA recruitment service. Participants received 1500HUF (approx. 4,60€) worth of shop vouchers as remuneration. All experiments in this study were approved by the United Ethical Review Committee for Research in Psychology (EPKEB). Participants provided written consent before taking part in the experiment.

#### Stimuli

Face stimuli were taken from the Karolinska Directed Emotional Faces (KDEF) stimulus set [34]. These stimuli were selected because they show a variety of faces in different head orientations and posing different expressions. Four identities were selected (2 male, 2 female; identities F9, F17, M9, and M12) and paired in same-sex configurations. We did not validate the stimuli we selected on the basis of emotional arousal or valence, nor did we select them on this basis. The reason for this was that, although emotion was a key manipulation in this study, faces in Experiments 1 and 3 were all shown with the same emotion, and were never shown in isolation. Thus, there was no ambiguity as to what emotion any individual face posed. Even in Experiment 2, where two different emotions shown at a time, these were shown repeatedly and consistently in the same pairings for long enough durations (500ms) as to remove any ambiguity. As such, the understandability of the emotional expressions could be easily derived from the experimental context even with the inclusion of expressions that would be otherwise ambiguous. In the interest of being thorough, however, we direct readers to Goeleven et al. [35], which includes observers’ accuracy in decoding each expression of each individual of the KDEF set (average accuracy across emotions: F9–0.74; F17–0.57; M9–0.56; M12–0.62).

The left and right half-profile images (head oriented 45° from centre) for each identity were used as stimuli–we used these face images so that directionality could be readily understood (engaged dyads could see each other whereas disengaged dyads could not) but enough of the face was still visible so as not to impair emotion recognition. Although this orientation does not give the impression of truly mutual gaze (faces looking directly at each other in foveal vision), faces in engaged dyads are nonetheless more engaged than are disengaged or catch trials, as their head orientation means that both of them can see each other, even if not in foveal vision, while for other trial types either one or both faces could unambiguously not see the other. Images were edited in Photoshop to add a blank white background that matched the experimental screen background. Face images were presented measuring approximately 5.1 x 6.3 degrees visual angle (0.18 x 0.4 normalised units; estimated viewing distance 55cm) and were displayed 3.4 degrees either side of the central fixation (0.12 normalised units).

#### Design and procedure

**Fig 1:**
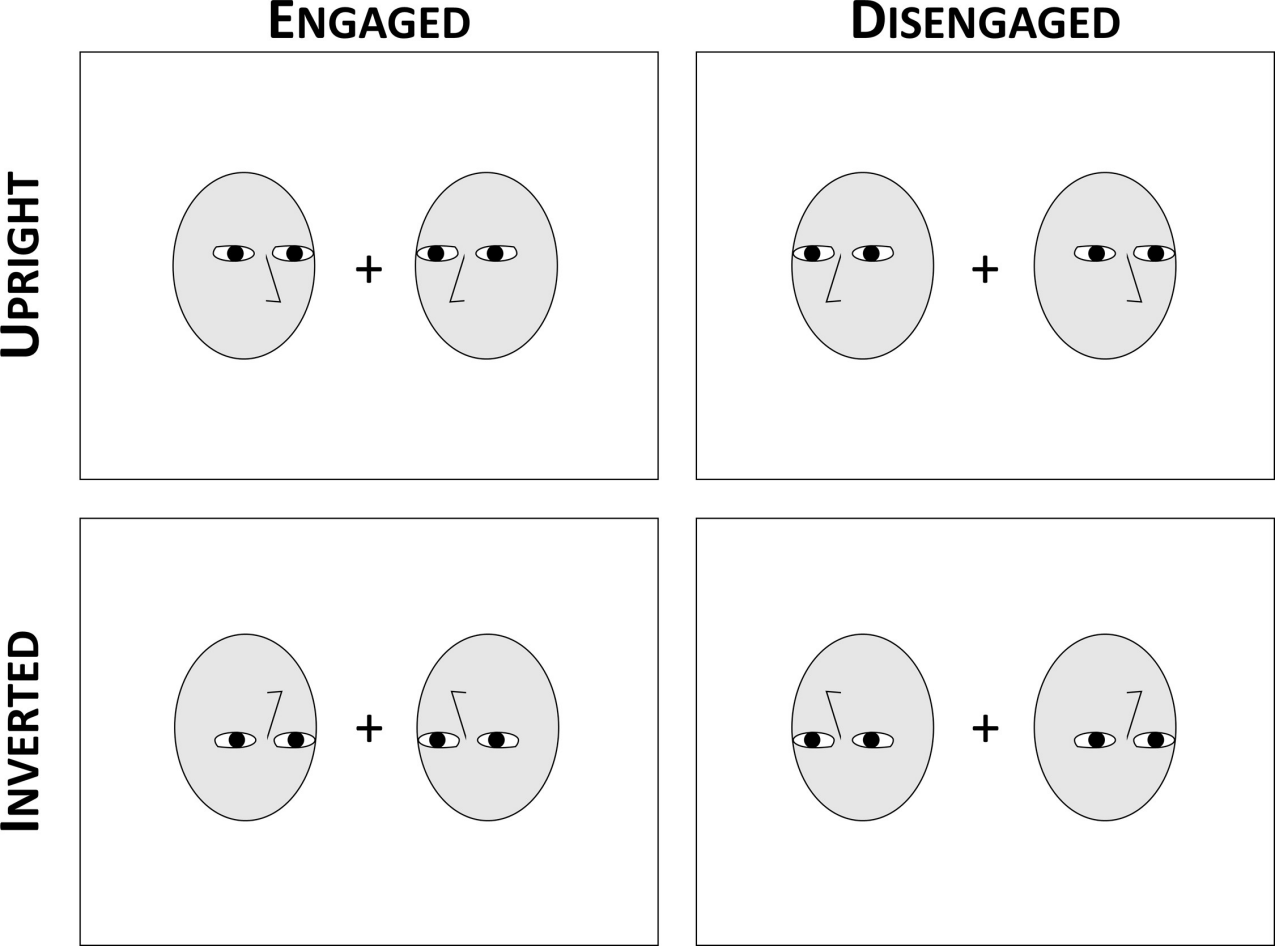
Example trials. Face pairs appeared in one of four configurations: engaged (left) or disengaged (right) and upright (top) or inverted (bottom). Schematic face examples are shown for illustrative purposes only and do not show the images that were used as stimuli in the experiment, which were half-left and half-right facing emotional images from the KDEF stimulus set [34].

The experiment was coded in PsychoPy 2.0 and ran fullscreen on a 2048x1152 monitor. Participants were told to make decisions about pairs of faces that appeared on the screen. Two faces were presented on either side of a central fixation cross. Participants were instructed to judge whether the pair was looking towards each other (engaged), or away from each other (disengaged). We chose to use head orientation judgements so that participants were making a task-relevant judgement (responding to a key feature of the experimental design) that could result in a 2-alternative forced choice. Although we are not aware of previous studies that look at effects of stimulus inversion on the judgements of head orientation in face pairs, pilot testing indicated that this manipulation and task were sufficient to generate reliable dyad inversion effects and perceptual grouping of engaged faces. Faces were presented upright on half of the trials, and vertically inverted on the other half. See Fig 1 for examples of all four trial conditions. Participants saw all faces posing either neutral, angry, or happy expressions.

Faces could look towards each other (an engaged pair) or away from each other (disengaged), and participants were instructed to respond with the keys A and L (mapping counterbalanced). 20% of trials were catch trials where both faces looked in the same direction (either left or right), on which participants were instructed to press the space bar. These catch trials were included so that participants could not answer the questions correctly by looking only at one face–previous research has shown that disrupting the mutual visual access within a dyad such that only one individual is facing the other eliminates the dyad inversion effect [16].

Trials began with a central fixation cross presented for 500ms. The faces then appeared for 500ms and then disappeared for a further 1,000ms. Participants could respond to the trial at any point within this 1,500ms window. A feedback screen was then shown, displaying a green tick mark for 1,000ms if participants were correct or a red X for 2,000ms if they were incorrect or missed a trial. This longer inter-trial interval was included as an incentive to encourage participants to be more accurate in the future.

Each face was presented on either side of fixation and looked left or right equally often. Each possible configuration (upright, inverted; engaged, disengaged) was repeated 20 times a block for 4 blocks, leading to 320 total trials. Participants also completed a first block that we designated a practice, during which the proportion of catch trials was increased to 33% (that is, face pairs were equally likely to both look in the same direction as to look either at each other or away). This was done so that participants could get used to making the 3-alternative choice.

#### Data analysis

We removed participants who scored below 70% accuracy on catch trials. This allowed us to remove participants who were not paying close enough attention to the stimuli, without removing or contaminating possibly interesting results in the analysis of accuracy rates. Four participants were excluded and replaced on this basis. Catch trials were not analysed further.

Reaction times (RTs) were analysed only on trials where correct responses were given, using ANOVA with RT as dependent measure. RTs were filtered to remove those values that were lower than 150ms or greater than 3 standard deviations above the mean of the given condition (e.g. a particular orientation, relationship, and emotion). This outlier analysis removed 157 trials overall (1.03%). We first ran a 2x2x3 mixed ANOVA with relationship and orientation as within-subjects factors, and emotion as a between-subjects factor. Given that the main test of our hypothesis was a stronger inversion effect for engaged dyads than disengaged dyads, we then performed a series of 2x2 repeated measures ANOVAs for each emotion separately. In this analysis, a significant interaction driven by a larger inversion effect for engaged than disengaged dyads would be taken as evidence of perceptual grouping.

We then supplemented this frequentist analysis with Bayesian one-way paired t-tests comparing the RTs to upright and inverted trials separately for engaged and disengaged faces for each emotion, which allows us to evaluate the strength of evidence for the alternative and null hypotheses of each inversion effect. For these t-tests we used uninformed priors and directed testing such that we expected RTs on inverted trials to be longer than RTs on upright trials.

Accuracy rates were generally very high in all experiments in this study, and for the sake of conciseness analyses of accuracy rates for all three experiments are reported in the Supplementary Material.

Data were analysed in JASP v.0.9.0.1.

### Results

**Fig 2:**
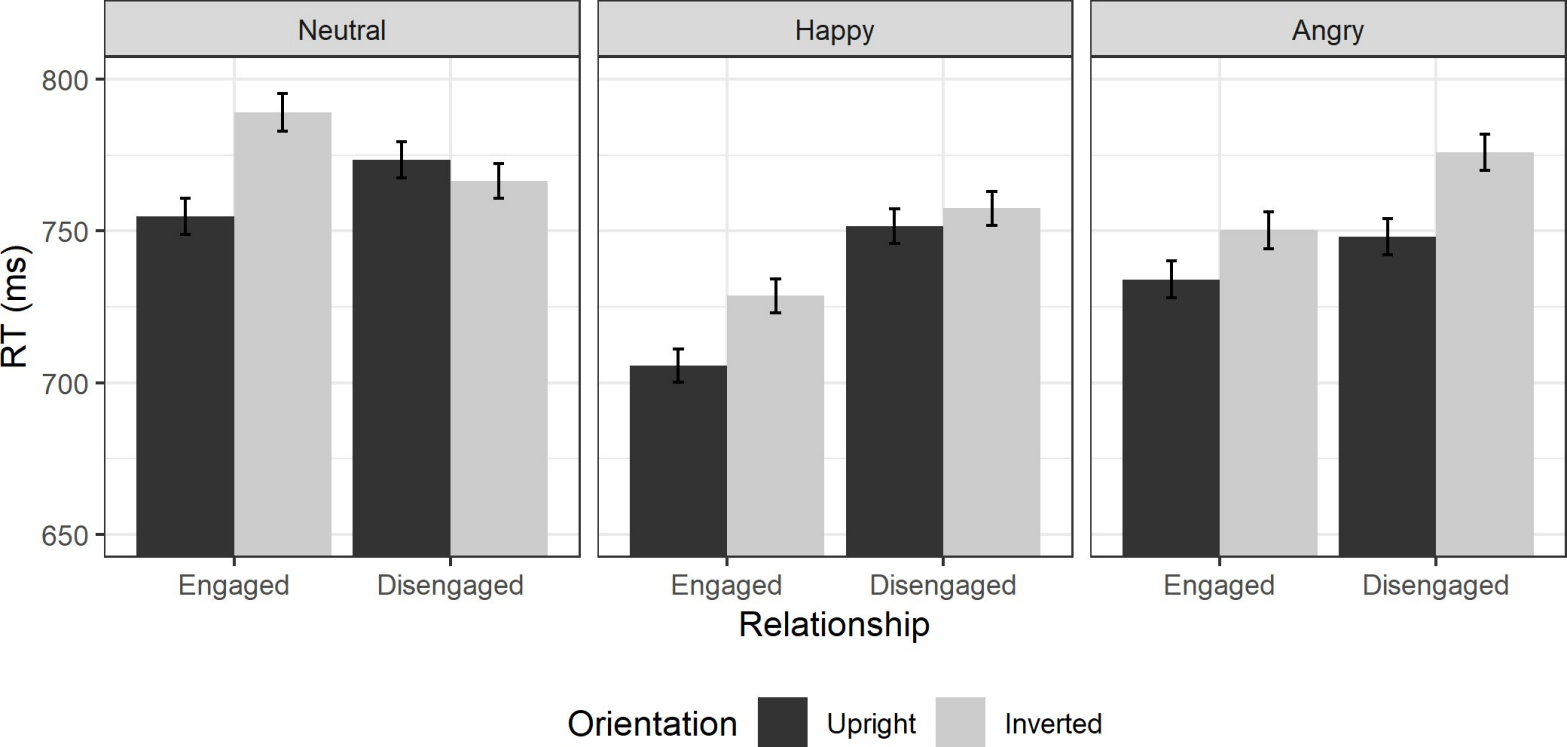
Experiment 1 Reaction times. Average reaction times in milliseconds in Experiment 1 to upright face pairs (dark grey bars) and inverted face pairs (light grey bars) for engaged (left) and disengaged configurations (right bars) to each emotion. Error bars show ±1 within-subjects standard error.

RTs from Experiment 1 are shown in Fig 2.

#### Frequentist ANOVA

The ANOVA found no significant main effect of emotion (F(2,45) = 0.56, p = .573, $\eta_{p}^{2}$ = 0.02), and no interaction of emotion with orientation (F(2,45) = 1.4, p = .272, $\eta_{p}^{2}$ = 0.06), or relationship (F(2,45) = 2.99, p = .060, $\eta_{p}^{2}$ = 0.12). However, there were significant main effects of relationship (F(1,45) = 7.72, p = .008, $\eta_{p}^{2}$ = 0.15), and of orientation (F(1,45) = 32.88, p < .001, $\eta_{p}^{2}$ = 0.42). There was also a two-way interaction of these factors (F(1,45) = 9.72, p = .003, $\eta_{p}^{2}$ = 0.18) and a three-way interaction with emotion (F(2,45) = 7.28, p = .002, $\eta_{p}^{2}$ = 0.24).

In a 2x2 repeated measures ANOVA looking at neutral faces, there was no main effect of relationship (F(1,15) = 0.04, p = .848, $\eta_{p}^{2}$ = 0.00). There was, however, a main effect of orientation (F(1,15) = 6.07, p = .026, $\eta_{p}^{2}$ = 0.29) and a significant interaction driven by a larger inversion effect for engaged than disengaged faces (F(1,15) = 15.25, p = .001, $\eta_{p}^{2}$ = 0.50). Follow-up t-tests (Bonf. α correction: 0.025) found that there was no significant inversion effect for disengaged faces (t(15) = -0.10, p = .539, d = -0.03) but there was for engaged faces (t(15) = 5.44, p < .001, d = 1.36).

For happy faces, there was a main effect of relationship, where engaged pairs were processed faster than disengaged pairs (F(1,15) = 10.00, p = .006, $\eta_{p}^{2}$ = 0.40), as well as a main effect of orientation (F(1,15) = 8.89, p = .009, $\eta_{p}^{2}$ = 0.37). There was also an interaction consistent with a perceptual grouping mechanism, as the inversion effect was larger for engaged dyads than disengaged (F(1,15) = 9.06, p = .009, $\eta_{p}^{2}$ = 0.38). Follow-up t-tests (Bonf. α correction: 0.025) found that there was no significant inversion effect for disengaged faces (t(15) = 0.95, p = .180, d = 0.24) but there was for engaged faces (t(15) = 4.21, p < .001, d = 1.05).

For angry faces, there was a main effect of orientation as RTs were faster to upright than to inverted dyads (F(1,15) = 22.49, p < .001, $\eta_{p}^{2}$ = 0.60). However, there was no effect of relationship (F(1,15) = 2.73, p = .119, $\eta_{p}^{2}$ = 0.15) and no interaction between the two (F(1,15) = 0.83, p = .378, $\eta_{p}^{2}$ = 0.05).

#### Bayesian contrasts

In order to supplement the frequentist analysis, we examined the nature of the interactions for each emotion separately with Bayesian paired samples t-tests. This allows us to go further than frequentist planned contrasts allows, as it allows us to evaluate the amount of evidence for the null hypothesis in cases where we do not expect to find an inversion effect.

We performed Bayesian t-tests looking at the RTs of upright and inverted face trials for engaged and disengaged faces separately. We used uninformed priors, and a directional hypothesis that RTs to inverted faces should be greater than RTs to upright faces.

For neutral faces, we found moderate evidence to support the null hypothesis for an inversion effect in disengaged faces (BF_10_ = 0.24). However, there was extreme evidence for the alternative hypothesis in engaged face pairs (BF_10_ = 795.87), indicating that there was indeed an inversion effect for engaged pairs that was absent for disengaged pairs.

For happy faces, we found weak (anecdotal, in Bayesian terminology) evidence for the null hypothesis for an inversion effect in disengaged pairs (BF_10_ = 0.61). However, we found very strong evidence for an inversion effect in engaged pairs (BF_10_ = 97.07).

For angry faces, we found a different pattern. There was extreme evidence for an inversion effect in disengaged face pairs (BF_10_ = 243.54) and moderate evidence for an inversion effect in engaged face pairs (BF_10_ = 8.49).

### Discussion

Experiment 1 aimed to generalise the dyadic inversion effect to faces, and to explore the role that emotional expression plays in this effect. We found a dyadic inversion effect for both neutral and happy face pairs. This suggests that dyadic inversion effects are not solely driven by the magnitude of the inversion effect that one would expect to see for an emotional face in isolation, as happy faces have frequently been shown to be subject to smaller inversion effects than angry faces [29–31]. For both happy and neutral faces, engaged pairs showed a strong to extreme canonical inversion effect, while disengaged pairs showed weak or anecdotal (happy faces) to moderate (neutral faces) evidence *against* an inversion effect. This suggests that, for neutral and happy expressions, orienting faces away from each other disrupted the perceptual grouping of dyads. For angry faces, however, we found evidence to support inversion effects in both engaged and disengaged face pairs. This indicates that perceptual grouping of individuals into pairs can be affected by the emotions expressed by the individuals in a dyad. These results raise the question of why dyadic inversion effects also occur when angry faces were looking away from each other.

One possibility is that angry faces are not subject to the same perceptual grouping mechanisms as neutral and happy faces. This is consistent with some evidence that angry faces are more difficult to perceptually group than are friendly faces [36]. As such, this suggests that the pattern of results associated with a perceptual grouping mechanism (a weaker inversion effect for disengaged pairs than engaged pairs) is a case where the presence of a salient configuration (engaged pairs not showing anger) overturns the default inversion that disengaged pairs would typically be subject to. As such, it is not that perceptual grouping leads to an inversion effect of engaged pairs, but that it disrupts the holistic processing of disengaged pairs that appear close to each other. When perceptual grouping is not possible (because angry faces are difficult to group), there may be a default that two faces appearing close together are subject to an inversion effect.

The pattern of results for angry faces is similar to the pattern Papeo et al. [7] found when they blurred the heads of bodies oriented towards and away from each other. When the head was obscured, they also found an inversion effect for both configurations rather than just for engaged pairs, although the inversion effect was still larger for engaged than disengaged pairs. In Experiment 1, we show similar inversion effects for engaged and disengaged pairs where head orientation is still available but faces show angry expressions. This could suggest that anger may have a similar disruptive effect on perceptual grouping of dyads as obscuring head information, perhaps because this expression transforms the apparent social relationship between identities such that they are no longer automatically considered a holistic unit.

Alternatively, it could be that whether different individuals are perceptually grouped depends on whether they form a meaningful configuration. Two faces looking angry while oriented away from each other may be considered socially meaningful, as two angry people may look away from each other in order to diffuse their emotions. It could be that the feature that drives this perceptual grouping is not the emotional expression per se, but the amount of information that can be readily inferred about the relationship between the faces, be it positive or negative in valence, which means that the meaningful disengaged configuration for angry faces is perceptually grouped in the same way as engaged pairs. Experiment 2 attempted to test this latter explanation. If the meaningfulness of a particular configuration is important, then the perceptual grouping seen in Experiment 1 should be stronger for emotions that, when engaged, are complementary (such as one person showing anger and the other showing fear [32,37,38]).

## Experiment 2

In order to examine whether the consistent inversion effects across both engaged and disengaged configurations with angry faces in Experiment 1 could be due to the meaningfulness of the relationships they implied, we ran a new experiment where faces posed two different emotions that were either complementary in their meaning (anger and fear) or that were not meaningfully complementary (in that they offered no inherent additional information about the social relationship beyond their constituent emotions) but had separately shown the effect in Experiment 1 (happy and neutral).

If the results of Experiment 1 were driven by the meaningfulness of the configurations, a complementary Anger-Fear combination of emotions should show a stronger perceptual grouping effect than a non-complementary Happy-Neutral combination. If, however, the results were driven by the presence of particular emotions (happy or neutral expressions) then the perceptual grouping effect should emerge more strongly for the Happy-Neutral combination than for the Anger-Fear combination. Finally, as Experiment 1 showed both faces posing the same emotion, we can test whether the symmetry of emotional expression is necessary for perceptual grouping effects to emerge.

### Materials and methods

#### Participants

A further 16 participants (10 female, M_age_ = 27.31yr) were recruited for this experiment in the same way as in Experiment 1. In this experiment, no participants were excluded on the basis of errors.

#### Stimuli, design and procedure

The basic procedure was the same as in Experiment 1. Participants still made decisions about the spatial relationship between the faces (engaged or disengaged) while these faces appeared in either upright or inverted configurations.

Rather than having both faces showing the same expressions, we presented pairs showing two emotions in combination–these emotions could either be complementary in terms of meaning (anger and fear) or not complementary in the sense that they afford no obvious interpretation beyond their spatial relationship (happy and neutral). In this experiment, rather than manipulating emotion between-subjects, participants saw both complementary and non-complementary emotion combinations. As such, in this experiment there were three two-level within-subjects factors: emotion complementarity, relationship, and orientation.

#### Data analysis

We retained the catch trial accuracy exclusion criterion detailed in Experiment 1. In this experiment, no participants had to be excluded on this basis.

RTs were once again filtered to remove outliers as in Experiment 1 (64 trials, 1.26% removed) and were analysed with frequentist three-way (including emotion complementarity as a factor) and two-way ANOVAs (separately for Anger-Fear and Happy-Neutral dyads) and then with Bayesian planned t-tests to evaluate evidence for the inversion effect in each combination of relationship and emotion complementarity.

### Results

**Fig 3:**
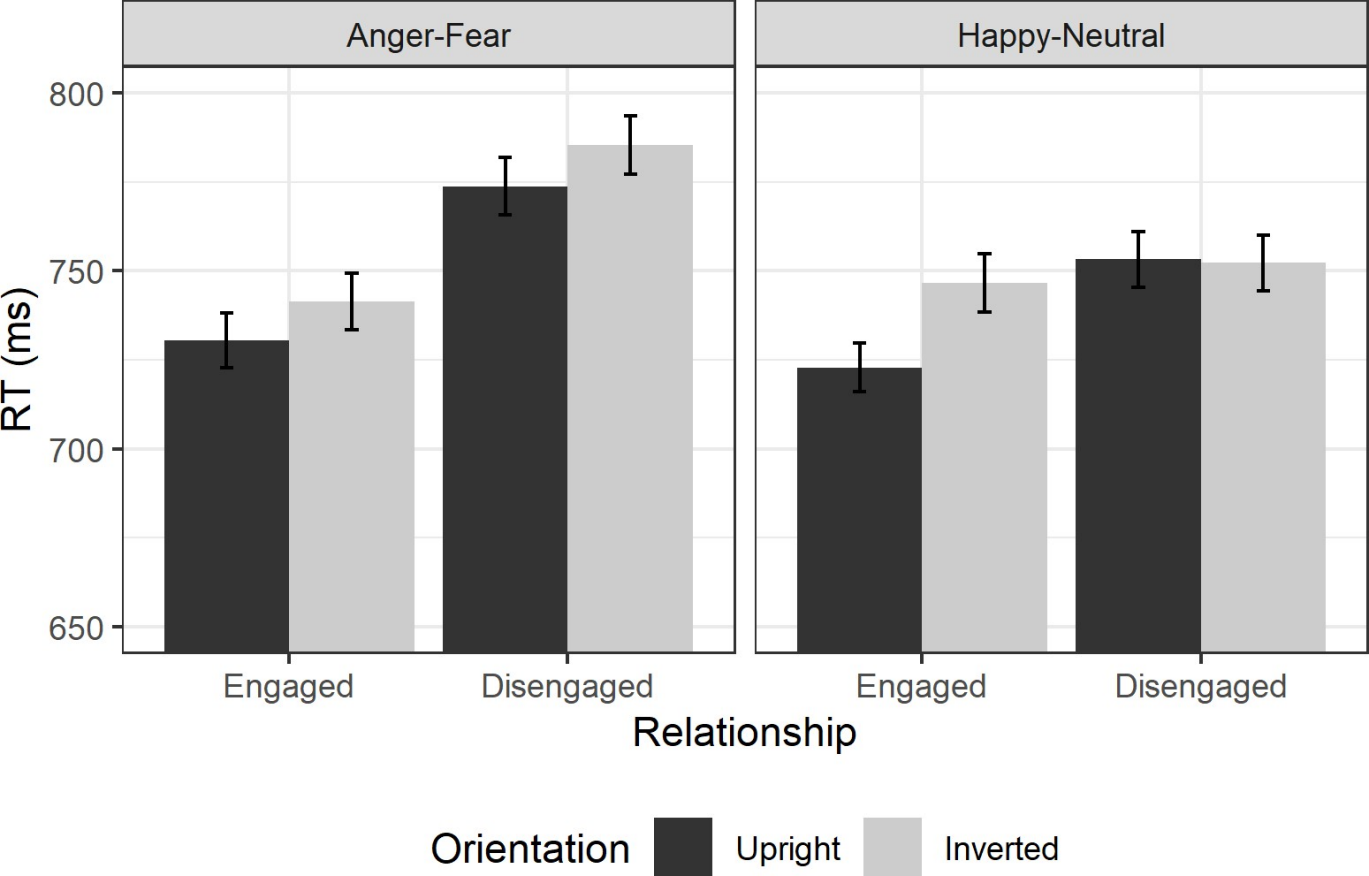
Experiment 2 Reaction times. Average RTs in Experiment 2 to upright face pairs (dark grey) and inverted face pairs (light grey bars) for engaged (left) and disengaged configurations (right bars) to Anger-Fear and Happy-Neutral emotion combinations. Error bars show ±1 within-subjects standard error.

The RTs of Experiment 2 are shown in Fig 3.

#### Frequentist ANOVA

RTs were analysed with a 2x2x2 within-subjects ANOVA using relationship, orientation, and emotion complementarity as repeated measures factors. The ANOVA found a significant main effect of relationship (F(1,15) = 9.88, p = .007, $\eta_{p}^{2}$ = 0.40), where responses were faster to engaged than disengaged pairs. Exploratory two-way t-tests found that for upright dyads RTs were significantly faster for engaged than disengaged dyads (Anger-Fear: t(15) = 3.12, p = .007, d = 0.78; Happy-Neutral: t(15) = 2.51, p = .024, d = 0.63), which was in the predicted direction. There was also a main effect of emotion (F(1,15) = 4.80, p = .045, $\eta_{p}^{2}$ = 0.24), but this was in the opposite direction from predicted, as participants were numerically slower to respond to Anger-Fear trials than Happy-Neutral trials on upright trials, although exploratory two-way t-tests did not find significant differences for either relationship (engaged: t(15) = 0.81, p = .430, d = 0.20; disengaged: t(15) = 1.37, p = .191, d = 0.34). There was an interaction between these two factors (F(1,15) = 7.01, p = .018, $\eta_{p}^{2}$ = 0.32) as the effect of relationship was more pronounced for Anger-Fear dyads than Happy-Neutral dyads.

There was no significant effect of orientation (F(1,15) = 1.41, p = .254, $\eta_{p}^{2}$ = 0.09), and orientation did not interact with either relationship (F(1,15) = 1.72, p = .210, $\eta_{p}^{2}$ = 0.10) or emotion (F(1,15) = 0.00, p = .997, $\eta_{p}^{2}$ = 0.00). The three-way interaction was also not significant (F(1,15) = 1.52, p = .236, $\eta_{p}^{2}$ = 0.09).

In a 2x2 repeated measures ANOVA looking at Anger-Fear dyads, there was a main effect of relationship (F(1,15) = 18.48, p < .001, $\eta_{p}^{2}$ = 0.55), where RTs were faster to engaged dyads than disengaged dyads. However, there was no main effect of orientation (F(1,15) = 1.29, p = .273, $\eta_{p}^{2}$ = 0.08) and no interaction between the two (F(1,15) = 0.11, p = .746, $\eta_{p}^{2}$ = 0.01).

For Happy-Neutral dyads, there was no main effect of relationship (F(1,15) = 2.95, p = .107, $\eta_{p}^{2}$ = 0.16), or of orientation (F(1,15) = 0.64, p = .438, $\eta_{p}^{2}$ = 0.04). Although the data did show the pattern of a perceptual grouping effect, in that the difference between upright and inverted dyads was larger for engaged dyads than for disengaged dyads, this interaction was not significant (F(1,15) = 4.35, p = .055, $\eta_{p}^{2}$ = 0.23).

#### Bayesian contrasts

Planned Bayesian contrasts were performed to test the inversion effect (slower RTs to inverted than upright trials) for each possible combination of relationship and emotion complementarity. These paired t-tests found weak (anecdotal) evidence for the null hypothesis for both engaged (BF_10_ = 0.75) and disengaged face pairs (BF_10_ = 0.35) when faces posed Anger-Fear emotions. For Happy-Neutral emotions, there was moderate evidence for the null hypothesis for disengaged pairs (BF_10_ = 0.19) and moderate evidence for an inversion effect in engaged pairs (BF_10_ = 4.42)–the signature of a perceptual grouping effect.

### Discussion

Experiment 2 aimed to test whether the perceptual grouping effect for socially engaged faces would be strengthened when these faces posed emotions that were complementary in terms of the meaningfulness of the apparent relationship between the faces (Anger-Fear) over when they were not meaningfully complementary but used emotions that have successfully shown the effect in the past (Happy-Neutral). We found that, rather than strengthen the perceptual grouping effect, Anger-Fear pairs showed no evidence of any inversion effects.

The perceptual grouping effect was present for pairs showing happy and neutral expressions, but this was a weak effect with only moderate evidence to support it. This could be due to the within-subjects design that we used in Experiment 2. While Experiment 1 manipulated emotion between-subjects, participants in Experiment 2 saw both combinations of emotional expression. It is possible that seeing four different emotions masked the perceptual grouping. It is also possible that emotional symmetry (that is, both faces posing the same emotion), while not necessary to induce perceptual grouping [14], does help to strengthen the effect.

Our results showed a cost associated with Anger-Fear trials, which was against our predictions. One explanation is that these dyads showed fear as an emotion. Fear, unlike happiness and anger, is an avoidance emotion–from an egocentric perspective, observing fear emotions leads to participants judging people as moving away rather than towards them [39]. It could be that someone expressing fear disrupts any perceptual grouping to a dyad as fear implies that the individual expressing fear is going to perform a flight response to some perceived threat. We tested this explanation in Experiment 3.

## Experiment 3

In Experiment 3 we used the same procedure outlined in Experiment 1 but with different emotions. In this experiment we included fearful expressions, this time posed by both faces in the dyad. If fear does disrupt perceptual grouping, then there should be no inversion effect for either engaged or disengaged face pairs.

Another aim of Experiment 3 was to replicate the effect seen in Experiment 1 with neutral and happy faces that was only partially replicated in Experiment 2. As such, we included neutral expressions to replicate the perceptual grouping effect.

A further emotion we tested in Experiment 3 was sadness. We included this emotion to see whether perceptual grouping effects are driven by valence. So far, the conditions that have failed to show perceptual grouping always conveyed negative emotional expression, rather than positive or neutral emotional expressions. If perceptual grouping only emerges for positive or neutral expressions, then there should be no dyadic inversion effect for sad faces.

### Materials and methods

#### Participants

We recruited 50 participants for this study, but due to high error rates on catch trials we excluded two, leaving 48 in total. There were 16 participants for each emotion. Due to a technical error demographic data were not collected for this experiment.

#### Stimuli, design, procedure, and data analysis

In this experiment we used the fear and sad expressions of the KDEF stimulus set instead of the happy and angry emotions. All other details of the paradigm and analysis plan were identical to Experiment 1. RT filtering removed 221 trials in total (1.21%).

### Results

**Fig 4:**
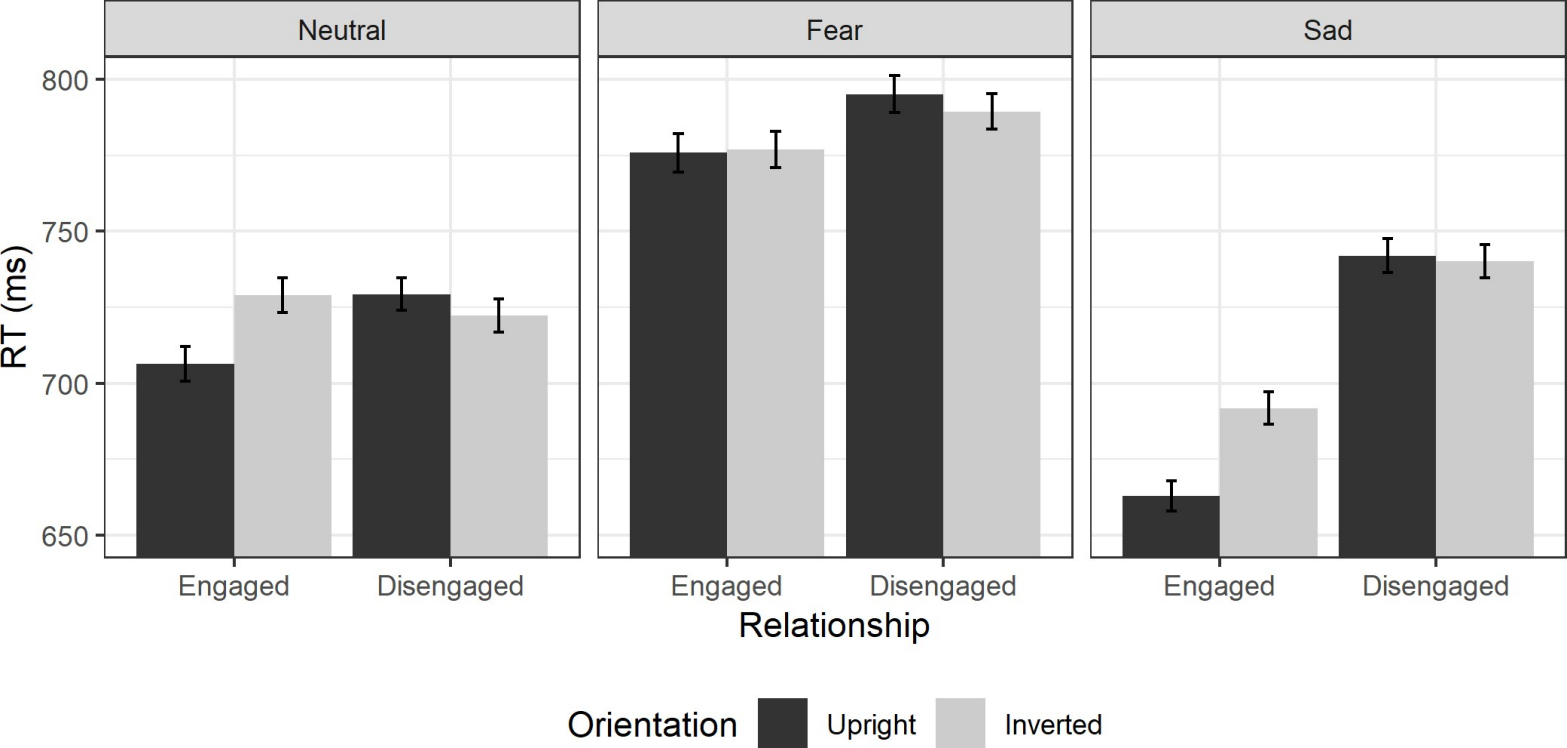
Experiment 3 Reaction times. Average RTs in Experiment 3 to upright face pairs (dark grey) and inverted face pairs (light grey bars) for engaged (left) and disengaged configurations (right bars) to each emotion. Error bars show ±1 within-subjects standard error.

RTs in Experiment 3 are shown in Fig 4.

#### Frequentist ANOVA

RTs were first analysed with a 2x2x3 mixed ANOVA with relationship and orientation as within-subjects factors, and emotion as a between-subjects factor. This ANOVA found a significant main effect of emotion (F(2,45) = 3.92, p = .027, $\eta_{p}^{2}$ = 0.15), but no significant interaction of emotion with orientation (F(2,45) = 1.08, p = .348, $\eta_{p}^{2}$ = 0.05). There was a significant interaction of emotion and relationship (F(2,45) = 3.39, p = .043, $\eta_{p}^{2}$ = 0.13), as the difference in RTs between engaged and disengaged dyads was stronger for sad faces than other emotions. There was a main effect of relationship (F(1,45) = 10.29, p = .002, $\eta_{p}^{2}$ = 0.19), but not one of orientation (F(1,45) = 3.05, p = .087, $\eta_{p}^{2}$ = 0.06). While there was a two-way interaction of relationship and orientation (F(1,45) = 10.24, p = .003, $\eta_{p}^{2}$ = 0.19), there was no significant three-way interaction with emotion (F(2,45) = 0.89, p = .419, $\eta_{p}^{2}$ = 0.04). As this indicates that evidence for perceptual grouping (an interaction of relationship and orientation) is not significantly different across the three emotions, separate 2x2 ANOVAs on each level of emotion are not necessary. However, the pattern of results shown in Fig 4 suggest that this two-way interaction of relationship and orientation is driven primarily by neutral and sad faces, while there seems to be little evidence that fear faces show evidence for a perceptual grouping effect. These 2x2 ANOVAs can help to clarify whether there are indications that the above interpretation could be valid.

In a 2x2 repeated measures ANOVA looking at neutral faces, there was no main effect of relationship (F(1,15) = 0.69, p = .418, $\eta_{p}^{2}$ = 0.04), nor was there a main effect of orientation (F(1,15) = 2.20, p = .159, $\eta_{p}^{2}$ = 0.13). While the pattern was broadly consistent with a perceptual grouping mechanism and similar to the results seen in Experiment 1, in this experiment the interaction was not significant (F(1,15) = 4.11, p = .061, $\eta_{p}^{2}$ = 0.22).

For fear faces, there was no main effect of relationship (F(1,15) = 0.91, p = .357, $\eta_{p}^{2}$ = 0.06), or of orientation (F(1,15) = 0.00, p = .970, $\eta_{p}^{2}$ = 0.00) and no interaction between the two (F(1,15) = 0.67, p = .426, $\eta_{p}^{2}$ = 0.04).

For sad faces, there was a main effect of relationship, as RTs were faster to engaged than disengaged dyads (F(1,15) = 11.60, p = .004, $\eta_{p}^{2}$ = 0.44). Although the main effect of orientation was not significant (F(1,15) = 4.06, p = .062, $\eta_{p}^{2}$ = 0.21) there was a significant interaction between the two (F(1,15) = 7.20, p = .017, $\eta_{p}^{2}$ = 0.32). Follow-up one-way t-tests (Bonf. α correction: 0.025) found that there was no significant inversion effect for disengaged faces (t(15) = -0.26, p = .599, d = -0.06) but there was for engaged faces (t(15) = 3.96, p < .001, d = 0.99).

#### Bayesian contrasts

We planned Bayesian contrasts to match Experiments 1 and 2. Given that this allows us to go further than frequentist planned contrasts would allow, as it enables us to evaluate the amount of evidence for the null hypothesis in cases where we do not expect to find an inversion effect.

We performed Bayesian t-tests looking at the RTs of upright and inverted face trials for engaged and disengaged faces separately. We used uninformed priors, and a directional hypothesis that RTs to inverted faces should be greater than RTs to upright faces.

For neutral faces, we found moderate evidence to support the null hypothesis for an inversion effect in disengaged faces (BF_10_ = 0.15). There was also moderate evidence for the alternative hypothesis in engaged face pairs (BF_10_ = 4.68), indicating that there was an inversion effect for engaged pairs that was absent for disengaged pairs.

For fearful faces evidence suggested there was no inversion effect for either engaged or disengaged pairs, and the evidence supported the null hypothesis for both engaged pairs (BF_10_ = 0.36, anecdotal) and for disengaged pairs (BF_10_ = 0.17, moderate).

For sad faces, we did see evidence of perceptual grouping. There was moderate evidence for the null in disengaged face pairs (BF_10_ = 0.21) but very strong evidence for an inversion effect in engaged face pairs (BF_10_ = 62.41).

### Discussion

The perceptual grouping for neutral faces observed in Experiment 1 was not fully replicated–although the same pattern was found, this was weaker and not significant in Experiment 3. Sad face dyads were also subject to perceptual grouping, as evidenced by a strong inversion effect for engaged face pairs and no inversion effect for disengaged pairs. However, fearful faces showed no inversion effects regardless of being engaged or disengaged. This finding indicates that fearful faces were not perceptually grouped in any configuration.

The finding that fearful dyads are not perceptually grouped provides further support for the hypothesis that perceiving fear (an avoidance emotion) disrupts the apparent social binding induced by mutualistic gaze that results in perceptual grouping. Vestner et al. [14] showed that engaged body pairs are judged to be standing closer to each other than are disengaged pairs (Experiment 2). On the other hand, people showing fear expressions are perceived as moving away more than those showing angry faces [39]. If fearful faces create an impression of avoidance in an allocentric perspective, and this perceived distance disrupts perceptual grouping, this could be tested using the distance illusion detailed in Vestner et al [14]. It could be an avenue for future research to investigate whether allocentric perception of spatial distance is affected by fearful expressions.

It is also worth noting that RTs were generally much longer to process fearful pairs than any other emotions. It could be that there is some processing window following stimulus presentation during which these perceptual grouping mechanisms emerge, and if RTs occur after this window then participants have been able to overcome the disruption associated with inversion. We find this explanation unlikely as Vestner et al. [14] showed perceptual grouping effects with RTs of over 1000ms (albeit in a visual search task rather than a direction judgement task).

## General discussion

We present the results of three experiments investigating perceptual grouping of emotional face dyads. When dyads both pose the same emotions, we find evidence of perceptual grouping for happy, neutral, and sad emotions. Angry emotions do not lead to preferential grouping for engaged faces and instead show an inversion effect for both engaged and disengaged pairs, while fear expressions disrupt all inversion effects, suggesting that fearful faces are not subject to any perceptual grouping. Pairs consisting of individuals showing different emotions show results consistent with their component emotions (perceptual grouping of Happy-Neutral pairs, no evidence for grouping of Anger-Fear pairs).

These findings contribute to a growing literature on whether and how two people are perceptually grouped into a single perceptual unit. This perceptual grouping mechanism, which is thought to reflect an early visual sensitivity to a particular spatial configuration, has been shown with bodies before. With this study, we show that it is also possible to replicate this perceptual grouping with faces alone, suggesting that head direction information is sufficient to elicit the effect in the absence of other body cues.

The dyad inversion effect has been shown to be robust across low-level differences in body postures [7] and body sizes [14]. The fact that this effect is preserved in these cases could be taken to suggest that this is a social effect–a sensitivity in the human visual system that is specifically tuned to a particular spatial configuration that conveys a certain type of social information, and so is sensitive to these configurations across a range of low-level noise. Our findings further support this by showing that this perceptual grouping mechanism is sensitive to the posed emotions of the faces involved, and that angry and fearful faces are not perceptually grouped in the same ways as happy, neutral, or sad faces. This suggests that this perceptual grouping mechanism is at least minimally sensitive to emotional information in these configurations.

We believe that our findings shed some light on the underlying processes of the dyadic inversion effect. Primarily, this effect seems to reflect a visual sensitivity to faces that are looking towards each other compared with faces looking away from each other, in a similar mechanism to the classic inversion effect. Inversion effects are typically interpreted as reflecting a visual sensitivity to a particular upright configuration–that is, there is a default prior within the visual system that face-like stimuli should appear with the eyes above the head. Inversion presents stimuli in a way that violates this default prior, leading to reductions in accuracy and response speed. Our findings suggest that there is a similar sensitivity to dyadic configurations–that is, there is a default prior within the visual system that two faces should appear oriented towards each other. When we presented dyads with neutral expressions that were oriented away from each other, we saw a similar cost in RTs to dyads that were presented inverted. The fact that these costs were not additive suggests that they reflect the same visual sensitivity mechanism–a default prior about how incoming sensory information should be configured. This sensitivity to faces is similar to the dyad inversion effect that has been observed with full body images [7,14–16]

Specific emotional expressions, then, could disrupt this mechanism–either by changing the perceived distance between the individuals or generally slowing down responses to the point that additional RT costs are not incurred (fear) or by making individual faces more difficult to perceptually bind (anger). The stronger evidence for perceptual grouping in Experiments 1 and 3, where all faces showed the same emotional expressions throughout, raises the possibility that this emotional consistency meant that emotion and the relationship between different faces’ emotions did not need to be computed on every trial–given that the emotional expressions were unambiguous and predictable, they could have been incorporated into participants’ prior expectations. The weaker evidence for perceptual grouping in Experiment 2 may reflect the additional interpretative load on participants as they had to recognise and decode facial expressions on every trial in order to interpret the relationship between faces, although clearly the question of how or whether mixed-emotion dyads are perceptually grouped remains an unanswered question and an avenue for future research.

This raises the question of how emotion affects this perceptual grouping, and whether it is the individual emotions of faces within the dyad that are important, or the relationship between these faces that are driving the effect. The importance of individual emotions is shown in Experiment 2 –even though Anger-Fear emotions are complementary and meaningful in terms of the relationship they convey, the dyad inversion effect was extinguished for these pairs likely due to the inclusion of fear as an emotion, which suggests that individual emotions are a driving feature of perceptual grouping. It is possible that, although emotions can change the nature of the apparent relationship between individuals, this is a computation that is beyond the capacity of this perceptual grouping mechanism. As Vestner et al. [14] show that engaged dyads are subject to attentional capture in a visual search paradigm, and Papeo et al. [7] show this grouping effect emerges with only a 30ms backwards-masked presentation, this suggests that this perceptual grouping mechanism of facing dyads is an early perceptual mechanism designed to detect a particular social configuration. While individual faces’ emotional expressions may aid or disrupt this attention prioritisation, it could be that deducing the emotional relationship between dyads is a later process that is not part of the perceptual grouping mechanism.

In the original two-body inversion effect published by Papeo et al. [7] the body avatar stimuli showed a variety of body postures. Body posture can also be used as a cue to emotional state in a similar way to facial expressions [40], but this was not directly controlled in that study. In the light of the current findings, it would be interesting to see whether apparent emotion from body posture affects this perceptual grouping in a similar way to facial expressions. If it does, then this supports the interpretation that this effect reflects a sensitivity to the apparent relationship between others. On the other hand, if body posture does not affect perceptual grouping in the same way as facial expression this could further highlight the importance of the head in this dyad inversion effect.

This study also raises questions about the impact of specific emotions on perceptual grouping of dyads. One such question is whether anger is a standout emotion in terms of how disengaged face pairs show inversion effects, and if so what the mechanisms for this may be. The fact that sad faces do show evidence of dyadic inversion rules out that this result with anger is driven by valence and suggests that anger may be a particularly disruptive emotion for the perceptual grouping effect. There is evidence that anger is a particularly salient emotion that is privileged in early perception [41]. It could be that this saliency disrupts perceptual grouping by encouraging processing of individual faces. Perceptual grouping leads to a cost in detecting local features, as the holistic representation is given priority over local features [42], and dyadic grouping compromises detection of particular individuals within that dyad [15]. It is possible that making the local features (the individual faces) more salient disrupts this grouping and encourages processing of individual faces over dyads [43]. While there is some evidence that particular emotions can affect higher-level cognitive processes such as decision making and belief evaluation [44], there is little work investigating whether these emotions have different effects on early visual processes like perceptual grouping.

Another outstanding question is why there was no perceptual grouping of fearful faces, and whether this reflects an allocentric decoupling mechanism or a processing time course for the effect. Again, as with angry faces this result does not seem to be due to emotional valence, as sad faces do show the expected dyad inversion effect. However, we cannot rule out that valence plays a role, perhaps interacting with another feature of the emotions such as arousal (sadness is a negative emotion, but it is not as arousing as either anger or fear, while happiness is arousing but not negative). Future research can investigate the role of interacting factors in this effect.

In conclusion, we successfully replicate the dyad inversion effect with faces and show that the perceptual grouping of engaged faces is affected by emotional expression, suggesting that the visual system goes beyond privileging the processing of a particular spatial configuration, and is sensitive to the emotional expression of individuals. This could be an early mechanism whereby particular individual emotions encourage dyadic grouping, which has downstream consequences for interpreting social, allocentric relationships between individuals in our environment. These results highlight the importance of studying the role of early visual processes in social scene perception.

## Supporting information

S1 File Analysis of accuracy rates from all three experiments. (DOCX)
