# Supplementary material for: The role of emotion in the dyad inversion effect
Source: PLoS One. 2019 Jul 2;14(7):e0219185. doi: 10.1371/journal.pone.0219185 (PMC6605658; doi:10.1371/journal.pone.0219185)
Supplement: S1 File — (DOCX) [file pone.0219185.s001.docx]

Supplemental Material

# Analysis of Accuracy Rates

Accuracy rates for all three experiments are shown in Supplemental Table 1. Accuracy rates were subjected to mixed effects logistic regression, with accuracy outcome (correct/incorrect) as dependent variable, emotion (happy/neutral/angry, Experiment 1; anger-fear/happy-neutral, Experiment 2; sad/neutral/fear, Experiment 3) as a between-subjects factor, and relationship (engaged/disengaged) and orientation (upright/inverted) as within-subjects factors. Participant identity was included as a random factor. As a planned follow-up for each experiment, we also compared the two-way relationship by inversion interaction for each between-subjects emotion separately in Experiments 1 and 3 (emotion was manipulated within-subjects in Experiment 2 and so is not broken down further). Data were analysed in *R* using the *glmer* function from the *lme4* package.

S1 Table. Accuracy as average proportion correct (with standard errors) for each condition across all emotions in Experiments 1, 2, and 3.

|  |  | Engaged | | Disengaged | |
| --- | --- | --- | --- | --- | --- |
|  |  | Upright | Inverted | Upright | Inverted |
| Experiment 1 | Neutral | 0.96 (0.01) | 0.94 (0.01) | 0.97 (0.01) | 0.96 (0.01) |
|  | Happy | 0.97 (0.01) | 0.95 (0.01) | 0.96 (0.01) | 0.96 (0.01) |
|  | Angry | 0.95 (0.01) | 0.93 (0.01) | 0.94 (0.01) | 0.95 (0.01) |
| Experiment 2 | Anger-Fear | 0.97 (0.01) | 0.96 (0.01) | 0.96 (0.01) | 0.96 (0.01) |
|  | Happy-Neutral | 0.96 (0.01) | 0.94 (0.01) | 0.96 (0.01) | 0.95 (0.01) |
| Experiment 3 | Neutral | 0.93 (0.01) | 0.92 (0.01) | 0.95 (0.01) | 0.96 (0.01) |
|  | Fear | 0.94 (0.01) | 0.93 (0.01) | 0.96 (0.01) | 0.97 (0.01) |
|  | Sad | 0.96 (0.01) | 0.94 (0.01) | 0.95 (0.01) | 0.94 (0.01) |

## Experiment 1

We analysed acccuracy rates with a three-factor logistic regression with emotion, relationship, and orientation as independent factors and accuracy (correct/incorrect) as a predicted variable. The only significant effect to emerge from this regression was a main effect of orientation, where accuracy was higher to upright than to inverted images (ß=-0.44, SE=0.20, z=-2.14, p=.033). No other main effects or interactions emerged (all zs<1.6). We also looked at the relationship by orientation interaction for each emotion separately.

For neutral faces, there was a significant effect of orientation (ß=-0.43, SE=0.20, z=-2.13, p=.033) but no effect of relationship (ß=0.22, SE=0.23, z=0.95, p=.342), and no interaction (ß=0.16, SE=0.31, z=0.53, p=.598). A similar pattern was found for happy faces: an effect of orientation (ß=-0.62, SE=0.24, z=-2.57, p=.010) but no effect of relationship (ß=-0.32, SE=0.25, z=-1.28, p=.201), and no interaction (ß=0.51, SE=0.33, z=1.54, p=.123). For angry faces, there was once again a main effect of orientation (ß=-0.47, SE=0.20, z=-2.36, p=.018) and no effect of relationship (ß=-0.27, SE=0.20, z=-1.34, p=.180), but there was a significant interaction (ß=0.60, SE=0.28, z=2.17, p=.030).

## Experiment 2

Descriptively, it seemed that there could be evidence of a perceptual grouping effect for Happy-Neutral trials, as the canonical effect (poorer accuracy on inverted trials) was there for engaged but not disengaged pairs. However, this difference was very small (~2.93% difference), and a general logistic regression found no effect of relationship (ß=-0.22, SE=0.33, z=-0.68, p=.499), orientation (ß=-0.06, SE=0.34, z=-0.18, p=.860) or emotion combination (ß=-0.27, SE=0.33, z=-0.84, p=.404) and no interactions between any two factors (all zs<0.9). The three-way interaction was also not significant (ß=0.34, SE=0.62, z=0.54, p=.587).

## Experiment 3

A three-factor logistic regression with emotion, relationship, and orientation as predictor variables found that there was no significant effect of relationship (ß=0.31, SE=0.18, z=1.77, p=.077) or orientation (ß=-0.20, SE=0.16, z=-1.28, p=.201). There was, however, a significant two-way interaction of relationship and orientation (ß=0.55, SE=0.26, z=2.15, p=.032). There was some evidence that sad emotions differed from neutral in terms of accuracy (ß=0.59, SE=0.32, z=1.82, p=.069), particularly for disengaged face pairs (ß=-0.46, SE=0.27, z=-1.70, p=.089), but these differences were not significant. No other effects or interactions were significant (all zs<1.3).

As in Experiment 1, we explored the results of the main analysis further with follow-up models for each emotion separately. For neutral faces, there was no significant effect of orientation (ß=-0.20, SE=0.16, z=-1.28, p=.200), nor was there a significant effect of relationship (ß=0.31, SE=0.18, z=1.77, p=.077), but there was a significant interaction (ß=0.55, SE=0.26, z=2.15, p=.032).

Fearful faces showed a significant main effect of relationship (ß=0.42, SE=0.19, z=2.19, p=.029) but no effect of orientation (ß=-0.14, SE=0.17, z=-0.85, p=.393), and no interaction (ß=0.45, SE=0.28, z=1.59, p=.112). For sad faces there was no main effect of orientation (ß=-0.31, SE=0.20, z=-1.59, p=.112), no effect of relationship (ß=-0.15, SE=0.20, z=-0.72, p=.469), and no significant interaction (ß=0.10, SE=0.27, z=0.35, p=.723).
